# The interplay of attachment styles and marital infidelity: A systematic review and meta-analysis

**DOI:** 10.1016/j.heliyon.2023.e23261

**Published:** 2023-12-03

**Authors:** Nasrin Ghiasi, Dara Rasoal, Arezoo Haseli, Rozhin Feli

**Affiliations:** a Department of Midwifery, School of Nursing and Midwifery, Ilam University of Medical Sciences, Ilam, Iran; b School of Health and Welfare, Dalarna University, Sweden; c Clinical Research Development Center, Motazedi Hospital, Kermanshah University of Medical Sciences, Kermanshah, Iran; d Student Research Committee, Kermanshah University of Medical Sciences, Kermanshah, Iran

**Keywords:** Anxiety, Attachment styles, Avoidance, Extramarital relation, Infidelity, Meta-analysis

## Abstract

**Objectives:**

Marital infidelity is a highly distressing experience for those involved. Understanding the psychological factors related to infidelity can help develop targeted interventions. The primary aim of this study was to assess the association between attachment styles and marital infidelity.

**Methods:**

Seventeen studies were included, sourced from electronic databases including PubMed, Scopus, Web of Science, and PsycInfo, with no time limitations, up to April 2023. The search employed terms like “attachment AND marital infidelity.” Study quality was evaluated using the Risk of Bias Assessment Tool from RevMan version 5.3.

**Results:**

The meta-analysis involved a total of 13,666 participants, ranging from 208 to 4047 individuals. Findings showed that higher levels of anxiety and avoidance in attachment were significantly associated with increased marital infidelity (*r* = 0.18, 95 % CI = 0.14–0.22, p < 0.0001). Conversely, weaker attachment insecurity was linked to reduced rates of marital infidelity. Additionally, both dismissive and fearful attachment styles correlated with marital infidelity, with respective weighted effect sizes of *r* = 0.07, p < 0.001 (95 % CI = 0.04–0.10) and *r* = 0.19, p < 0.001 (95 % CI = 0.10–0.29). No association was found between preoccupied attachment and infidelity.

**Conclusion:**

Individuals with insecure attachment styles, specifically those with high levels of anxiety or avoidance, are more likely to engage in marital infidelity. Attachment styles should be a focus in couples therapy, especially for treatment related to infidelity. Assessing and addressing these underlying attachment issues can better guide therapists in their work with couples facing infidelity.

## Highlights

• Numerous studies have explored the link between attachment styles and marital infidelity.
• Both anxiety and avoidance in attachment are strongly associated with marital infidelity.
• Dismissive and fearful attachment styles also correlate with instances of marital infidelity.
• While preoccupied attachment shows no significant relationship with infidelity.

## 1 Introduction

Marital infidelity poses a significant threat to the stability of romantic relationships [1]. Statistics from 2016 reveal that 20 % of older Americans admitted to extramarital relations, compared to 14 % among those under 55 years old [2]. The negative repercussions of infidelity extend beyond relationship dissolution [3], it is also the most prevalent cause of divorce and separation across various cultures and societies [4]. Furthermore, both parties involved in infidelity frequently experience detrimental psychological effects, such as increased risk of depression and guilt [5,6]. The ability to identify psychological traits correlated with a higher likelihood of infidelity could be instrumental for targeted interventions [1]. In this regard, attachment theory offers a useful framework [7,8]. According to this theory, individuals form mental schemas about the reliability of their close relationships, which in turn influence their behavioral and cognitive responses [7]. This framework has emerged as a potent tool for understanding relational dynamics within societal and personality psychology [9]. Previous studies have described that one's attachment style—defined by their willingness to engage in close relationships—has a significant impact on the quality of romantic relationships [10]. Specifically, insecure attachment styles have been linked to a greater likelihood of engaging in infidelity [11]. For example, individuals scoring high on anxious attachment or displaying preoccupied or dismissive attachment styles are more prone to engage in extramarital affairs [12]. However, while attachment theory provides a robust framework for understanding relational dynamics, it is argued that might oversimplify the complexities inherent in human relationships by attributing infidelity primarily to insecure attachment styles [13]. The discourse surrounding attachment styles and infidelity often overlooks other crucial factors such as relationship satisfaction, personal values, and external influences, all of which can play significant roles in the manifestation of infidelity [14].

Despite these advancements, a gap remains in the academic discourse, marked by divergent findings and interpretations. The primary objective of the present study is to clarify the association between attachment styles and marital infidelity. To this end, we conducted a systematic review and meta-analysis to comprehensively assess the association between adult attachment styles and infidelity.

## 2 Materials & methods

### 2.1 Eligibility criteria, information sources, and search strategy

This review adheres to the guidelines provided by the Preferred Reporting Items for Systematic Reviews and Meta-Analysis Protocol (PRISMA-P) [15] and the Cochrane Handbook for Systematic Reviews [16]. We conducted a comprehensive search in English across multiple databases up to April 2023, including PubMed, Scopus, Web of Science, PsycINFO, and the Cochrane Database of Systematic Reviews. We employed the following search query: “attachment AND (infidelity OR extra-marit* OR affair OR cheating OR unfaithful* OR disloyal* OR betrayal OR adultery OR ‘sexual activity outside marriage’ OR ‘extradyadic involvement')." In line with the definition of infidelity *as “a violation of a couple's assumed or stated contract regarding emotional and/or sexual exclusivity*” our search encompassed all forms of infidelity—sexual, emotional, combined sexual and emotional, and internet-based.

Eligibility Criteria.

To be included in this review, studies had to meet the following criteria.

(a) Investigate the relationship between adult attachment style and marital infidelity.
(b) Be published in English.
(c) Be formally published as peer reviewed articles (unpublished studies, books, and book chapters were excluded). Conference abstracts, reviews, and editorials were also excluded
(d) Present data directly relevant to the research hypotheses
(e) Utilize measures that evaluate attachment style in adults' close romantic relationships.
(f) Provide sufficient data for computing effect sizes.

### 2.2 Study selection

Two researchers independently searched electronic databases, collected potential studies, and sorted them according to the previously mentioned eligibility criteria. Duplicate entries were eliminated. The initial screening process involved reviewing titles and abstracts. Any ambiguities that arose were addressed by a third researcher who conducted in-depth assessments of the studies in question. Studies that met the inclusion criteria were selected through discussion and consensus within the research team. Finally, we documented relevant details and summarized the key findings.

### 2.3 Data extraction

The following information was extracted from each study that met the inclusion criteria.

- Author(s) and year of publication
- Study methodology
- Sample characteristics, including details such as sample size, gender distribution, sexual orientation, mean age, average relationship length, education level, ethnicity, and location (see Table 1 for details)

**Table 1:** Characteristics of studies and participants.

	Study	Assessment of attachment style	Assessment of infidelity	n	Male %	Participant (engage romantic relationship)	Heterosexual %	Mean age (SD)	Mean length of relationship (SD)	college degree%	Caucasian %	location
1	Pereira 2014 (16)	ECR-S	The Infidelity Scale	345	25	College students	None	19.46 (1.92)	None	100	N	United States
2	McDaniel 2017* (17)	ECR-S	7 items	338	48.8	married/cohabiting couples	None	F; 31.59(4.44) M; 33.26(5.05)	10.02 y (4.05)	73	92	United States
3	Fish 2012 (18)	ECR-R	The Infidelity Scale	353	26.3	General population	92.4	24	1-3 y	64.6	60.3	United States
4	Fricker 2006 (19)	ECR-R	The Infidelity Scale	312	20.2	General population	None	31.29 (11.88)	7 y (8.26)	86.3	N	Australia
5	Russell 2013 (20)	ECR-R	2 items	614	50	newlywed couples	None	F:23.5(3.8) M:24.9(4.4)	1–6 m	N	>90	United States
6	Ferron 2016 (21)	ECR-R	2 items	502	45.8	User computer	91.4	29.85 (9.91)	6.36 y (6.57)	76.5	N	Quebec
7	Stewart 2017 (22)	ECR-R	The Infidelity Scale	310	12	General population	78.9	18 to 84*	None	60	71	United States
8	Burchell 2011 (23)	ECR-R	1 item	437	31.8	General population	None	F:26.52(9.36) M:26.35(8.35)	None	None	N	Australia
9	Levy 2010 (24)	RSQ	Buss Infidelity Questionnaire	416	23.8	General population	None	26.6 (7.5)	None	None	23.0	United States
10	Amidon 2008 (25)	RSQ	Experiences of Infidelity 21 items	388	19.3	General population	94.8	22 (4.65)	14.3 m (39.80)	21.9	71.1	United States
11	Treger 2011 (26)	RSQ	7 items	3879	35.6	None	None	20**	None	None	85.3	United States
12	Tagler 2011 (27)	RSQ	2 items	489	27	Adults College students	88.8	43.52 (12.06)	15.01 y (12.02)	100	95.5	United States
13	Schmitt 2015 (28)	RSQ	Relationship Exclusivity scale 7 items	4047	36.5	None	None	None	None	None	66	United States
14	Owen 2013 (29)	AAS	1 item	252	25.4	General population	96.1	28***	1.49 y (1.21)	88.5	85.7	United States
15	Altınok 2020 (30)	RSQ	Intentions towards Infidelity Scale	407	43.5 %	university students	None	21.52 (3.58)	None	100	None	Turkey
16	Girard 2020 (31)	ECR-R	7 opened end Q	208	65 % female and 34 % male	university students	80 %	≥18	–	100	69 %	United States
17	Soltanzadeh 2021 (32)	AAS	The Attitude to Infidelity Questionnaire:	369	51 % male 39 % female	married students	100	26.8 < 25 43 % 25-30 29.6 % > 30	–	100	0	Iran

ECR-S: The Experiences in Close Relationships- Short form (12 Items); ECR-R: The Experiences in Close Relationships-Revised (36 Items); RSQ: Relationship Scale Questionnaire; AAS: The Adult Attachment Scale (AAS; Collins & Read, 1990); F: Female; M: Male; y: year; m: mount; *: age range; **: modal age; ***: median ag.
- Instruments used to measure attachment styles and infidelity

For data abstraction, the first author calculated effect sizes for each study included in the meta-analysis. Many studies presented correlational results, which were used as the effect size metric. In cases where means, standard deviations, and sample sizes were available, these were converted to correlation coefficients *r*. Additionally, when other specific data points were available—such as sample size, *r*, p-value, standard error (SE), or 95 % confidence intervals—at least two of these were input into the RevMan 5.3 software to further analyze the results.

### 2.4 Quality assessment

The quality of the included studies was evaluated using the Risk of Bias Assessment Tool from RevMan version 5.3, developed by the Cochrane Collaboration. This tool is designed to assess the extent to which studies have minimized methodological bias and is widely recognized for its reliability and feasibility [17]. Two researchers (AH and RF) independently assessed the quality of each study. Any discrepancies were resolved by a third (NGh) and fourth author (DR). Consensus was reached on various aspects, including the study objectives, clarity in describing the participant selection process (inclusion criteria), adequacy of the sample size, handling of missing data, and adherence to ethical considerations or participant consent.

### 2.5 Meta-analysis

We conducted a meta-analysis to describe the correlation between attachment styles and marital infidelity. The data analyses and syntheses were carried out using RevMan Version 5.3 software. If a study had not reported the Standard Error, we contacted the study's author to obtain this information. Heterogeneity among the studies was assessed using the *Tau*^*2*^ and *I*^*2*^ statistics. I^2^ values of 25 %, 50 %, and 75 % were considered indicative of low, moderate, and high levels of heterogeneity, respectively [18]. Both random-effects and fixed-effects meta-analyses were performed, depending on the level of heterogeneity. Specifically, random-effects meta-analyses were conducted when heterogeneity exceeded 50 %, while fixed-effects meta-analyses were employed when it did not. Attachment styles were assessed in the studies using three different scales: the Experiences in Close Relationships (ECR) scale, the Adult Attachment Scale (AAS), and the Relationship Scale Questionnaire (RSQ). Studies were categorized into five subgroups based on these scales and their respective subscales. The ECR and AAS scales feature two subscales for attachment styles: anxiety and avoidance. The RSQ scale includes four subscales: secure, preoccupied, dismissive, and fearful.

## 3 Result

### 3.1 Study flow

**Fig. 1:**
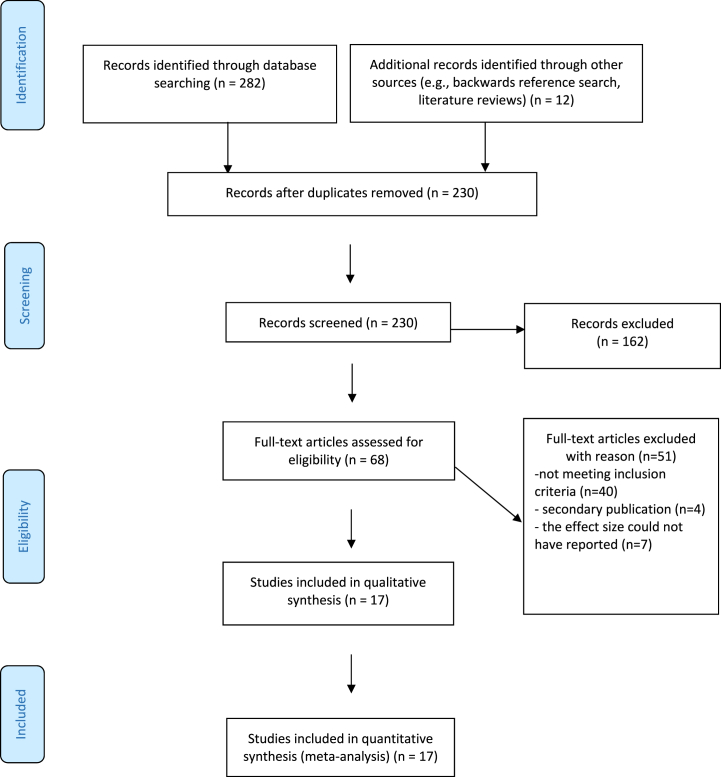
PRISMA flow diagram of the screening procedure.

Fig. 1 provides a flowchart outlining the progression of studies included in the current meta-analysis. Initially, a total of 294 records were screened. After the removal of 64 duplicate studies, 162 were immediately excluded for not meeting the inclusion criteria. Of the remaining 68 articles, full evaluations were conducted to assess their suitability for inclusion. Ultimately, 17 studies met all the inclusion criteria. The remaining 51 studies were excluded for the following reasons: 40 did not meet the inclusion criteria, 4 were secondary publications, and 7 lacked sufficient data to report effect sizes, as they did not conduct tests examining the correlation between attachment and infidelity.

### 3.2 The quality assessment (risk of bias)

**Fig. 2:**
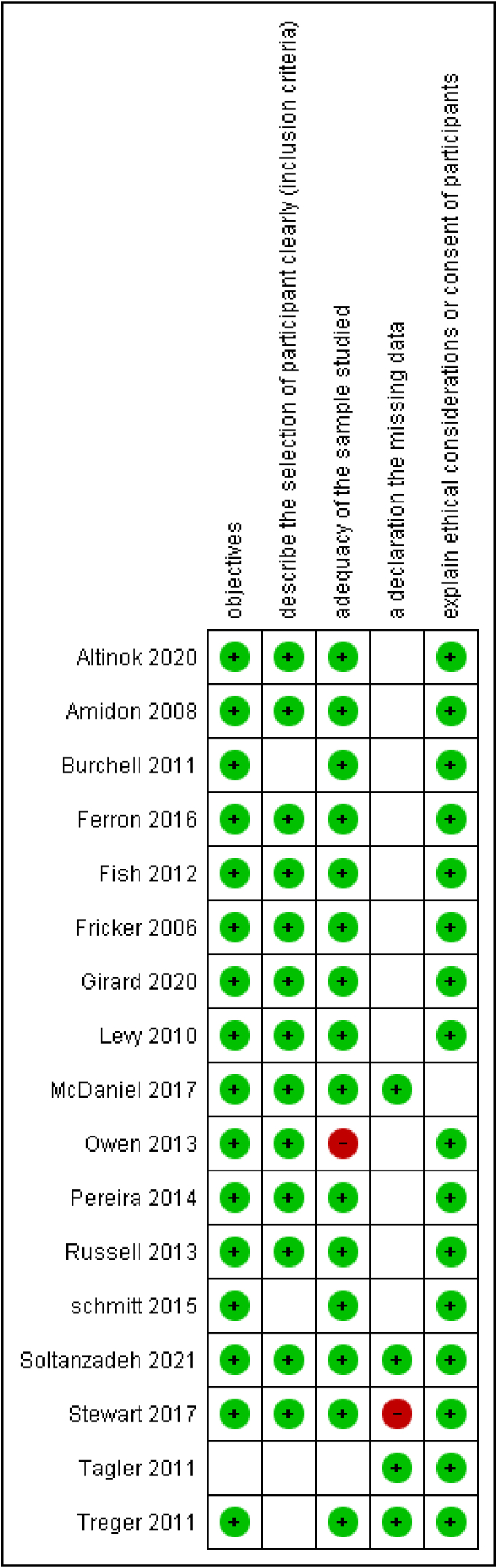
Summary of Risk of Bias – This figure illustrates the assessments of the review authors regarding each risk of bias item for each study included in the analysis.

The authors assessed the risk of bias in the study methods and categorized them into three groups: low risk, high risk, or unclear risk, in accordance with the Cochrane Collaboration tool. These classifications are fully detailed in Fig. 2.

### 3.3 Study characteristics

Relevant information from the 17 selected studies is summarized in Table 2, Table 3. The meta-analytic sample included a total of 13,666 participants, ranging from 208 to 4047 individuals per study. Table 1 outlines the characteristics of the studies and participants for each independent sample. Participants across all studies included both men and women: 35.61 % (n = 4866) were men, 62.66 % (n = 8563) were women, and 1.73 % (n = 237) had unspecified gender. A majority of studies (57.1 %) did not report any information on sexual orientation, but the remaining studies primarily featured heterosexual participants. Geographically, 12 studies were conducted in the United States, 2 in Australia, 1 in Canada, 1 in Turkey, and 1 in Iran. The mean length of relationships was reported in 8 studies (47.06 %) and ranged from 6 months to 15.01 years. Most participants were of Caucasian ethnicity. Regarding the instruments used, the Experiences in Close Relationships (ECR) questionnaire was employed in 7 studies (with 3 using the short form [19] and 5 using the revised form), while the Relationship Assessment Questionnaire (RAQ) [20] was used in 7 studies [21]. Additionally, two studies used the Adult Attachment Scale (AAS). Infidelity was assessed using The Infidelity Scale in nine studies, while the remaining studies used tools developed by the researchers.

**Table 2:** Random Effects Meta-Analysis of Attachment and infidelity with The Experiences in Close Relationships Scale.

Authors	n	Anxiety					Avoidance				
		r	95 % confidence interval		Z-value	p-value	r	95 % confidence interval		Z-value	p-value
			Lower limit	Upper Limit				Lower limit	Upper Limit		
Pereira 2014	345	0.129	0.00	0.26	1.96	0.05	0.263	0.06	0.46	2.58	0.01
McDaniel 2017^a^	338	0.24	0.11	0.36	3.37	<0.001	0.26	0.14	0.38	4.24	<0.001
Fish 2012	353	0.165	0.03	0.3	2.57	0.01	−0.19	−0.3	−0.07	−3.29	0.001
Fricker 2006	312	0.22	0.05	0.4	2.58	0.01	0.3	0.07	0.5	2.57	0.01
Russell 2013	614	0.74	0.017	0.18	3.34	0.001	−0.15	−0.67	0.37	−0.57	0.57
Ferron 2016	502	0.11	0.18	0.19	2.58	0.01	0.13	0.002	0.03	2.57	0.01
Stewart 2017	310	0.11	0.03	0.19	2.58	0.01	−0.027	−0.14	0.08	−0.49	0.63
Burchell 2011	437	0.252	0.06	0.44	2.6	0.01	0.188	0.05	0.3	2.61	0.01
Owen 2013	252	−0.19	−0.34	−0.045	−2.57	0.01	−0.08	−0.66	0.51	−0.27	0.79
Girard 2020	208	0.804	0.250	1.358	4.322	0.001	0.074	−0.003	0.151	1.883	0.597
Soltanzadeh 2021	369	0.450				<0.05	0.348				<0.05
*Weighted Mean r*	***3733***	***0.19***	***0.12***	***0.26***	***5.28***	***<0.001***	***0.19***	***0.13***	***0.25***	***6.37***	***<0.0001***

a Reported gender base but computed.

**Table 3:** Random Effects Meta-Analysis of Attachment and infidelity with Relationship Scale Questionnaire.

Authors	n	Dismissing					Preoccupied					Fearful				
		r	95 % confidence interval		Z-value	p-value	r	95 % confidence interval		Z-value	p-value	r	95 % confidence interval		Z-value	p-value
			Lower limit	Upper Limit				Lower limit	Upper Limit				Lower limit	Upper Limit		
Amidon 2008	388	0.06	−0.01	0.13	1.64	0.1	0.14	0.03	0.25	2.57	0.01	0.16	0.04	0.28	2.65	0.01
Schmitt 2015^a^	4047	0.09	0.05	0.13	4.41	<0.001	0.001	−0.019	0.02	0.1	0.92	0.05	0.03	0.07	4.90	<0.001
Tagler 2011	489	0.45	0.11	0.79	2.57	0.01	0.21	−0.34	0.76	0.75	0.45	0.07	−0.34	0.48	0.33	0.73
Levy 2010	416	0.35	0.00	0.70	1.95	0.05						0.28	0.111	0.446	3.29	0.001
Treger 2011	3879						0.42	0.11	0.72	2.7	0.007					
Altınok 2020	407	0.04					0.10	0.00	0.20		0.05	0.02				
*Weighted Mean r*	***9626***	***0.08***	***0.02***	***0.14***	***2.82***	***0.005***	***0.12***	***−0.01***	***0.25***	***1.78***	***0.08***	***0.14***	***0.02***	***0.25***	***2.27***	***0.02***

a Reported gender base but we computed it.

### 3.4 Quantitative data synthesis

**Fig. 3:**
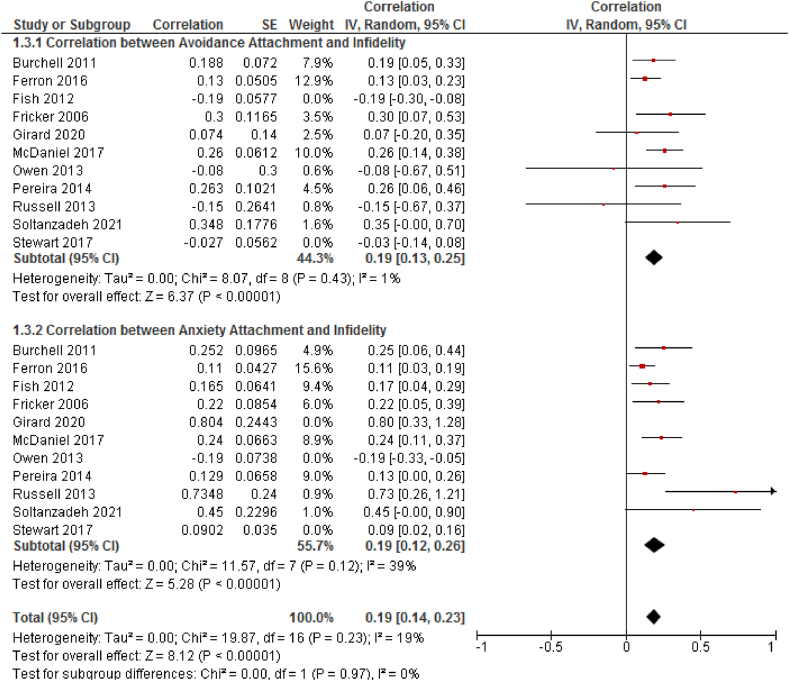
The result is a forest plot for correlation between attachment styles (based on ECR scale) and marital infidelity: Diamond sign indicate combination of all studies with 95 % confidence intervals.

**Overall Effect Size:** As hypothesized, higher levels of attachment anxiety and avoidance were associated with higher rates of infidelity. Conversely, lower levels of attachment insecurity were correlated with lower rates of infidelity. The overall weighted effect sizes were r = 0.19, P < 0.001 (95 % CI = 0.12–0.26) for attachment anxiety and r = 0.19, P < 0.001 (95 % CI = 0.13–0.25) for attachment avoidance, as illustrated in Fig. 3.

**Fig. 4:**
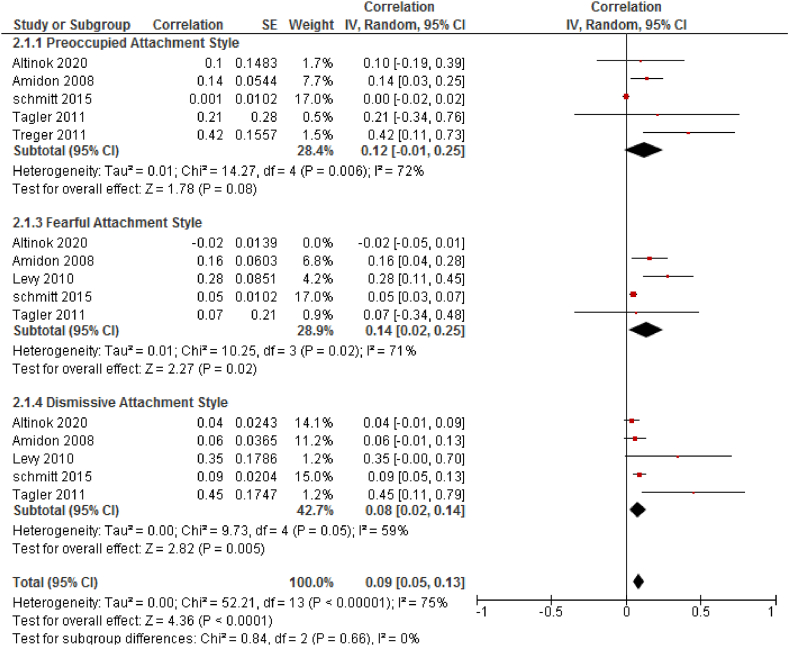
The result is a forest plot for the correlation between attachment styles (based on ARQ) and marital infidelity: Diamond sign indicate combination of all studies with 95 % confidence intervals.

Additionally, higher levels of dismissive and fearful attachment styles were predictive of infidelity. The overall weighted effect sizes were r = 0.08, P < 0.005 (95 % CI = 0.02–0.14) and r = 0.14, P = 0.02 (95 % CI = 0.02–0.25), respectively. In contrast, the preoccupied attachment style did not significantly predict infidelity, with an overall weighted effect size of r = 0.12, P = 0.080 (95 % CI = −0.01–0.25) (Fig. 4).

In the first subgroup analysis, which focused on the subscales of ECR and AAS, the initial heterogeneity for adult avoidance attachment was high (I^2^ = 79 %). This dropped to moderate levels (I_2_ = 1 %) after the exclusion of two studies [22,23]. Similarly, the initial heterogeneity for adult attachment anxiety was also high (I^2^ = 76 %) but decreased to low levels (I_2_ = 19 %) after excluding three studies [[23], [24], [25]]. The overall heterogeneity test showed low levels of heterogeneity (I_2_ = 19 %, P < 0.001), justifying the use of a fixed-effect model for this part of the meta-analysis (Fig. 3). In the second subgroup analysis, which was based on the subscales of ARQ, the overall heterogeneity was high (I_2_ = 75 %). The heterogeneity in the fearful subgroup reduced from 87 % to 0 % after the exclusion of two studies; however, the overall heterogeneity remained high. Due to the limited number of studies in each subgroup (dismissive, fearful, and preoccupied attachment styles), a random-effect model was utilized for this part of the meta-analysis. The heterogeneity of the included articles (*Tau*^*2*^ = 0.00) was deemed acceptable (Fig. 4).

## 4 Discussion

The current study aimed to systematically review the literature on the relationship between attachment styles and marital infidelity. Our findings suggest that both anxiety and avoidance attachment styles are related to marital infidelity.

### 4.1 Avoidant attachment style and infidelity

Individuals with an avoidant attachment style tend to exhibit a more promiscuous socio-sexual orientation, which may lessen their inclination to engage exclusively in sexual activities with their partner [26]. This attachment style is also linked to a preference for short-term sexual relationships [27], likely because these individuals find long-term commitments uncomfortable. Furthermore, people with avoidant attachment often demonstrate less interest in seeking emotional intimacy through sexual behavior and exhibit various indicators of dishonest behavior [28]. It's crucial to note that while avoidant individuals may shy away from deep emotional connections, they still need and benefit from support and closeness, typically seeking and providing it during periods of lower emotional distress [29]. A study by De Wall et al. encompassing eight different studies indicated that an avoidant attachment style is consistently associated with lower levels of commitment to one's romantic partner, which, in turn, predicts a greater interest in alternative partners and a higher likelihood of marital affairs [30].

### 4.2 Anxiety attachment style and infidelity

Individuals with anxious attachment styles are often characterized by an unconscious inclination to merge emotionally with their partners, whom they tend to idealize. However, because the responsiveness of their partners is often unclear, these individuals usually exhibit behaviors of neediness and clinging. They often struggle with low self-esteem and live with a constant fear of abandonment [29,31]. Reports from anxiously attached individuals indicate feelings of low self-worth, a belief that their partners do not love them sufficiently, and intermittent negative emotions. Consequently, they also report lower levels of happiness, trust, interdependence, and commitment in their relationships [14].

### 4.3 Dismissive and fearful attachment styles and infidelity

Additionally, the results of the current study suggest that individuals with dismissive and fearful attachment styles are more likely to engage in infidelity than those with a preoccupied attachment style. Both fearful and dismissive individuals fall under the ‘umbrella’ of avoidant attachment styles [32]. Bartholomew posits that there are actually two types of avoidant adults: those in the dismissing category, who exhibit disinterest in forming close relationships (dismissing-avoidant), and those in the fearful category, who partly fear close relationships (fearful-avoidant) [33]. Thus, it can be concluded that the results of this meta-analysis are consistent across both scales.

Fearful adults harbor an internalized sense of unworthiness and are highly reliant on external validation for their self-worth. Their negative view of others compels them to avoid intimacy for fear of potential rejection, despite their yearning for relationships. On the other hand, dismissive adults have negative expectations of others but positive views of themselves. Like their fearfully avoidant counterparts, they tend to avoid close friendships [34]. Insecurely attached individuals who exhibit high levels of avoidance, such as those with fearful or dismissive attachment styles, display distinct patterns in romantic preferences and behaviors [35]. They generally show less interest in committed relationships [36], and they report lower levels of relationship satisfaction, higher frequency of divorce [37], and reduced emotional closeness [38].

In support of these findings, one meta-analysis has shown that insecure attachment is strongly correlated with all types of sexual violations, including sexual infidelity, even in the absence of psychopathology [39]. Chopik et al. argue that attachment is a crucial component of one's life ‘from the cradle to the grave,’ suggesting that attachment continues to influence individuals throughout their lifespan [40]. Given that infidelity violates the closeness of romantic relationships and that romantic love encompasses sexuality, caregiving, and attachment [36], a connection between infidelity and attachment in intimate relationships seems plausible.

The statistical power of the current meta-analysis based on the Relationship Scale Questionnaire (RSQ) is lower than that based on the Experiences in Close Relationships (ECR) measure, primarily because fewer studies have utilized the RSQ (only 6 studies). Most of the research has been conducted in the United States, which may limit the generalizability of the study results. However, the findings of this meta-analysis suggest that despite potential variations in ethnicity and social factors, the relationship between attachment style and relevant predictor variables remains consistent [41,42].

### 4.4 Limitations and strength

This study has several limitations, which are present at both the study and review levels. First, there were few primary and homogeneous studies, affecting the overall analyses and particularly the subgroup analyses. Second, given that most of the early studies were conducted in the United States, the generalizability of these findings to other cultures remains uncertain. Third, although we identified a relationship between attachment styles and infidelity, the correlational nature of the studies prevents us from determining causality.

A notable strength of this meta-analysis is the high diversity of included studies in terms of infidelity measures, population demographics, and relationship lengths, which reflects the current state of the literature. However, this diversity could potentially mask important differences across studies, especially when statistical heterogeneity is not fully explained. It's worth noting that after excluding two outlying studies, the heterogeneity was fully accounted for, bolstering the reliability of our review since the results were consistent across a diverse range of populations. Additionally, meta-analyses can increase the statistical power of individual studies and facilitate exploratory analyses, thereby generating hypotheses for future research [43].

## 5 Conclusion

The current study aimed to systematically review the literature on the relationship between attachment styles and marital infidelity. Our findings suggest that both anxiety and avoidance attachment styles are related to marital infidelity. In conclusion, this study underscores the critical role of understanding insecure attachment styles to address marital infidelity effectively within therapy. The deep insights obtained facilitate personalized therapeutic strategies and empower couples to improve their relational dynamics, promoting healthier communication and mutual respect. These insights significantly impact the broader discourse on marital satisfaction and relationship dynamics, encouraging continued research in this vital area. The ultimate goal of therapy is to aid in fostering relationships marked by enhanced understanding, resilience, and lasting fulfillment.

## 6 Future research

Future research should not only focus on how self-oriented attachment styles may predict infidelity, but also examine the attachment styles of both partners, thereby emphasizing the need for a dyadic approach to relationships. Given that infidelity can serve as a psychological shock, which some researchers refer to as ‘attachment injury’ [44,45], future studies are encouraged to explore changes in attachment styles following such betrayals. Additionally, ongoing research is crucial for identifying personal characteristics that influence infidelity. This will enable therapists to more effectively address the specific needs of clients who seek therapy for issues related to infidelity.

## Ethical consideration

This study was part of a research project approved by the Ethics Committee of Research Deputy at Kermanshah University of Medical Sciences in Kermanshah (Ethical code: IR. KUMS. REC.1402.124).

## Funding

The present study was supported by Student Research Committee, 10.13039/501100005317Kermanshah University of Medical Sciences, Kermanshah, Iran (Grant number: 50002915).

## CRediT authorship contribution statement

**Nasrin Ghiasi:** Writing – review & editing, Writing – original draft. **Dara Rasoal:** Writing – review & editing, Methodology, Supervision, Validation. **Arezoo Haseli:** Writing – review & editing, Writing – original draft, Methodology, Data curation, Conceptualization. **Rozhin Feli:** Writing – original draft, Methodology, Data curation.

## Declaration of competing interest

The authors declare that they have no known competing financial interests or personal relationships that could have appeared to influence the work reported in this paper.
